# Supplementary material for: Deletion of wfs1 Impairs Oligodendrocyte Precursor Cells Dorsal Distribution and Myelination Through the wfs1-hmgcs1 Axis in Zebrafish
Source: Biology (Basel). 2026 Aug 16;15(16):1402. doi: 10.3390/biology15161402 (PMC13510151; doi:10.3390/biology15161402)
Supplement: Supplementary file 1 [file biology-15-01402-s001.zip › File S1-Transcriptome sequencing data and differentially expressed genes (DEGs) in wfs1-- zebrafish at 5 dpf/1.GO/wfs1KOvsWT/ALL/wfs1KOvsWT.all_GOdot.pdf]

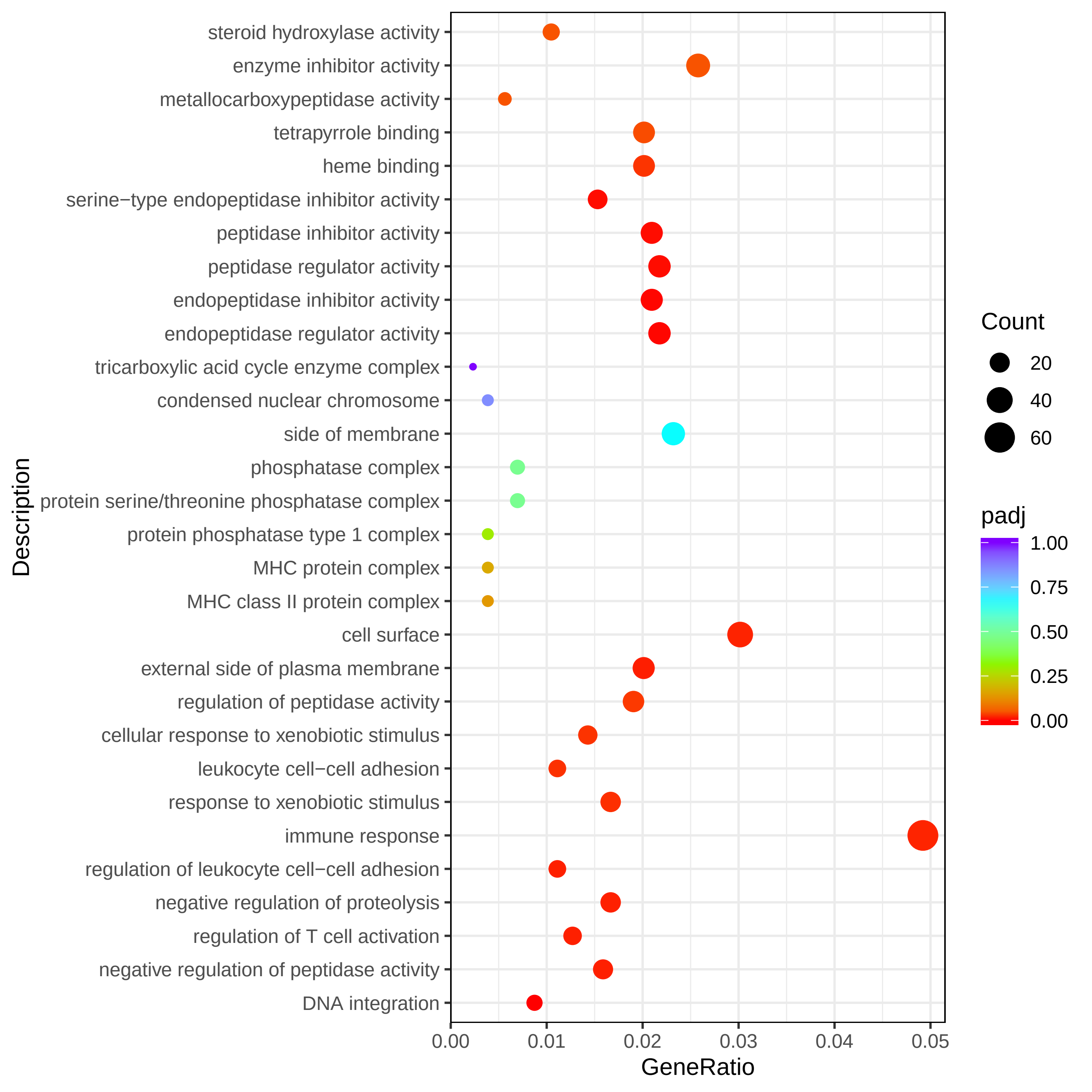
steroid hydroxylase activity
enzyme inhibitor activity
metallocarboxypeptidase activity
tetrapyrrole binding
heme binding
serine−type endopeptidase inhibitor activity
peptidase inhibitor activity
peptidase regulator activity
endopeptidase inhibitor activity
endopeptidase regulator activity
tricarboxylic acid cycle enzyme complex
condensed nuclear chromosome
side of membrane
phosphatase complex
protein serine/threonine phosphatase complex
protein phosphatase type 1 complex
MHC protein complex
MHC class II protein complex
cell surface
external side of plasma membrane
regulation of peptidase activity
cellular response to xenobiotic stimulus
leukocyte cell−cell adhesion
response to xenobiotic stimulus
immune response
regulation of leukocyte cell−cell adhesion
negative regulation of proteolysis
regulation of T cell activation
negative regulation of peptidase activity
DNA integration
Description
0.00
0.01
0.02
0.03
0.04
0.05
GeneRatio
Count
20
40
60
padj
1.00
0.75
0.50
0.25
0.00
